# Associations of TAP1 genetic polymorphisms with atopic diseases: asthma, rhinitis and dermatitis

**DOI:** 10.18632/oncotarget.23458

**Published:** 2017-12-20

**Authors:** Rongzeng Liu, Xiafei Chen, Jingjiao Qi

**Affiliations:** ^1^ Department of Immunology, Medical College, Henan University of Science and Technology, Luoyang, China; ^2^ Network Information Center, Henan University of Science and Technology, Luoyang, China

**Keywords:** TAP1, polymorphism, atopic diseases, atopic dermatitis, allergic rhinitis, Immunology

## Abstract

Controversial findings have been reported regarding to the effect of the transporter associated with antigen processing 1 (TAP1) polymorphisms exerted on the atopic diseases susceptibility. To gain a better understanding of the effects of TAP1 polymorphisms on the risk of atopic diseases, a retrospective study was carried out to evaluate the association of the most common TAP1 polymorphisms, rs1057141 and rs1135216, with the risk of atopic diseases. From studies published in PubMed, Embase, and Web of Science up to July 2017, ten eligible studies were selected for meta-analysis. The pooled results from rs1135216 polymorphism showed increased risk of atopic diseases in homozygote and recessive comparison. From the subgroup analysis by ethnicity, it was found that rs1135216 polymorphism contributed to atopic diseases susceptibility among Africans in all the five genetic models. Subgroup analysis by atopic types indicated significant association of TAP1 polymorphism rs1135216 with asthma in the allele, dominant and recessive models and with allergic rhinitis in the recessive model. As to rs1057141, increased risk of atopic disease in the allelic, dominant and heterozygous model was found in African population. Overall, this meta-analysis study demonstrated that rs1135216 polymorphism may contribute to atopic diseases susceptibility in Asians and Africans as assessed in this study. However, well designed large-scale case-control studies are needed to confirm such preliminary findings.

## INTRODUCTION

Atopic diseases which include atopic dermatitis(AD), allergic rhinitis(AR), and asthma(AA) are closely related. Allergic rhinitis is often present in asthmatics and the incidence of asthma development in individuals suffering from allergic rhinitis is five times of those bearing no allergic rhinitis [1]. It has been reported that allergic symptoms could start with atopic dermatitis in infancy followed by allergic rhinitis and asthma later in life [2]. In addition, development of atopic diseases is attributed to genetic and environmental factors in a great extent [3].

It has been proposed that TAP gene may be responsible for atopic disease susceptibility. Polymorphisms in TAP1 may affect antigen recognition and presentation, potentially resulting in low expression or entirely absence of MHC-I at the cell surface, which could ultimately lead to an adverse immune response [4]. Studies on the polymorphisms of the TAP1 gene in several human leukocyte antigen (HLA)-associated diseases such as atopic dermatitis, asthma, rhinitis [5–14] found that rs1057141 and rs1135216 as the most common single nucleotide polymorphisms. Currently, controversial opinions are presented for the relationships between TAP1 gene polymorphisms and atopic diseases. This may be resulted from the moderate sample size of the previous studies which could not provide inclusive findings. Therefore, to gain a more comprehensive insight of the association of TAP1 polymorphisms with the development of atopic diseases, our study performed a set of meta-analysis by utilizing pooled data from selected studies on the association of two TAP1 polymorphisms rs1057141 and rs1135216 and atopic diseases.

## RESULTS

### Study characteristics

**Figure 1:**
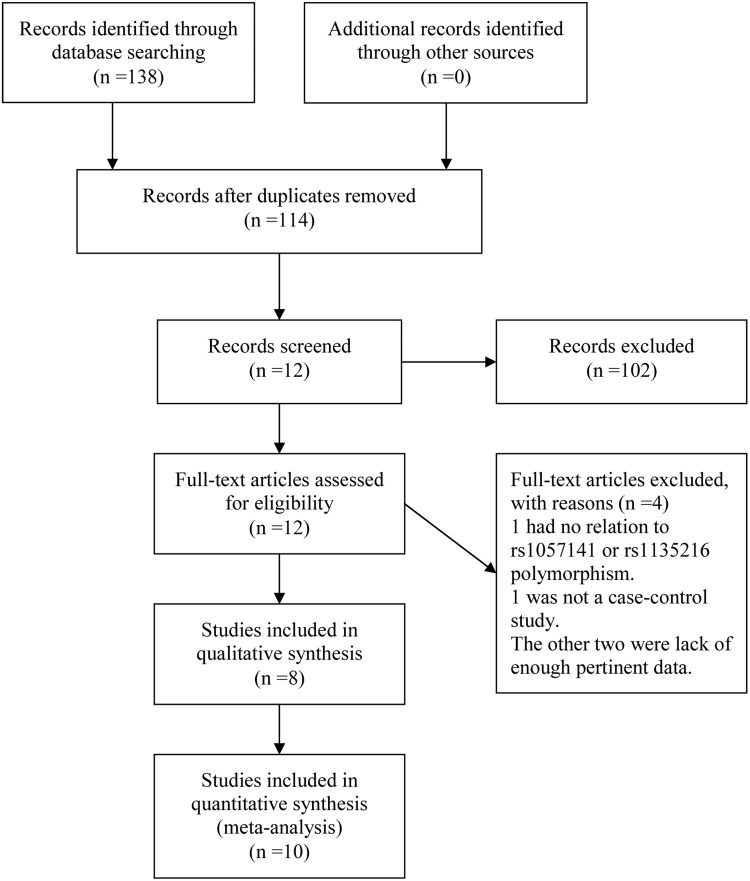
Workflow for literature selection

By following literature selection process as illustrated in Figure 1, 138 studies were identified from PubMed, Embase and Web of Science databases (PubMed: 20, Embase: 30, Web of Science: 88). Subsequently, 126 articles were excluded due to duplication (n=24) or without relation to this topic (n=102). Following detailed evaluation of the remaining twelve studies, four studies were excluded with one being lack of relation to rs1057141 or rs1135216 polymorphism [15], one not being a case-control study [16], and two lack of enough pertinent data [8, 14]. A total of 8 articles, with 7 published in English and 1 in Chinese, were included for qualitative synthesis based on the inclusion and exclusion criteria [5–7, 9–13]. One of the articles was further separated into three studies because it examined different atopic types [7] (Table 1). Therefore, in total, data from 10 case-control studies, amounting to 661 cases and 876 controls for rs1057141 and 668 cases and 876 controls for rs1135216, were pooled into the meta-analysis. Of these, there were 4 studies of atopic dermatitis, 4 studies of allergic rhinitis, and 2 studies of asthma. Characteristics of studies investigating the relationship between rs1057141 and rs1135216 single nucleotide polymorphism and atopic diseases are summarized in Table 1. For all the studies, genomic DNA isolated from blood samples was used for genotyping, for which PCR-ARMS assay were used in five studies; PCR-RFLP method were applied in three studies, PCR-SSCP assay was used in one study, and SNaPshot system assay was used in one study.

**Table 1:** Characteristics of studies in the meta-analysis

Author	year	Location	Ethnicity	Atopic type	Genotyping	TAP1 rs1057141 Case/Control				TAP1 rs1135216 Case/Control				NOS
						AA	AG	GG	HWE	AA	AG	GG	HWE	
Chen	2012	China	Asia	AR	SNaPshot	96/99	50/45	4/6	0.756	104/107	43/41	3/2	0.379	11
Kim	2007	Korea	Asia	AR	ARMS	82/54	26/53	2/0	0.001	76/55	30/50	4/2	0.014	8
Hang	2003	Taiwan	Asia	AA	RFLP	58/25	45/15	6/3	0.719	76/37	39/6	1/0	0.623	10
Takeuchi	2002	Japan	Asia	AR	ARMS	50/50	9/11	1/1	0.666	46/42	12/19	2/1	0.482	11
Lee	2001	Korea	Asia	AD	SSCP	34/113	16/60	3/11	0.427	36/125	15/55	2/4	0.469	9
Ismail	1997	Tunisia	Africa	AA	ARMS	31/65	15/14	2/2	0.261	19/62	6/16	23/3	0.159	10
Ismail	1997	Tunisia	Africa	AR	ARMS	24/65	21/14	0/2	0.261	19/62	15/16	11/3	0.159	10
Ismail	1997	Tunisia	Africa	AD	ARMS	12/65	6/14	2/2	0.261	8/62	9/16	3/3	0.159	10
Kuwata	1995	Japan	Asia	AD	RFLP	27/38	9/12	1/2	0.413	28/39	8/11	1/2	0.302	8
Kuwata	1994	Japan	Asia	AD	RFLP	21/28	8/7	0/0	0.511	26/22	3/13	0/0	0.177	8

AD, atopic dermatitis; AR, allergic rhinitis; AA, asthma; ARMS, amplification refractory mutation system; RFLP, restriction fragment length polymorphism; SSCP, single-strand conformation polymorphism; HWE, Hardy-Weinberg Equilibrium; NOS, Newcastle-Ottawa Scale.

Meta-analysis results

#### TAP1 rs1057141 polymorphism and atopic diseases susceptibility

**Figure 2:**
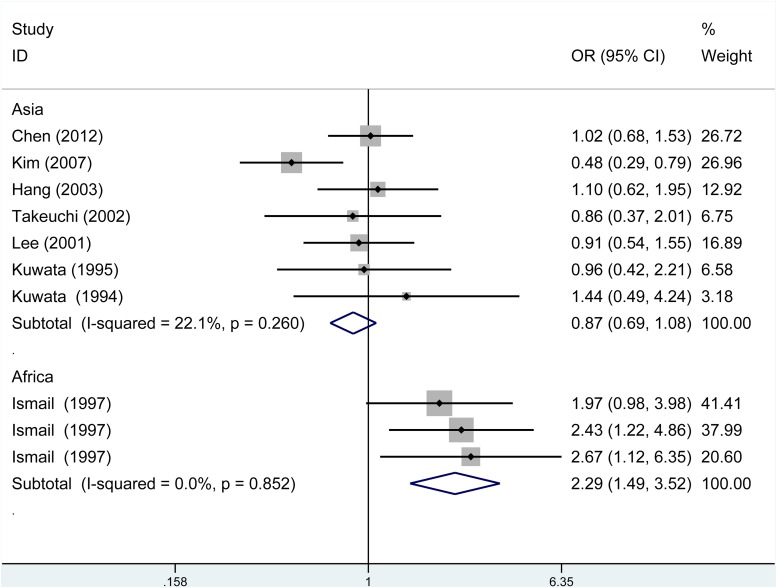
Subgroup analysis of the relationship between TAP1 rs1057141 polymorphism and atopic diseases stratified by the ethnicity of study population I^2^ and P_heterogeneity_ for the outcomes of the allelic comparison in Asians (I^2^=22.1%, *P*_h_=0.260) and Africans (I^2^ = 0, *P*_h_= 0.852).

The summary of meta-analysis for TAP1 rs1057141 polymorphism with atopic diseases was demonstrated in Table 2. Overall, significant association was not identified in any genetic model. Besides, it was revealed that significant heterogeneity was present in allelic, dominant, heterozygous genetic models (Table 2). Interestingly, when stratified by ethnicity, heterogeneity disappeared in Asians (I^2^ = 22.1%, *P*_h_ = 0.260) and Africans (I^2^ = 0%, *P*_h_ = 0.852) in allelic comparisons (Figure 2). In African population, increased risk of atopic disease was found in the allelic (odds ratio (OR)= 2.29, 95% CI (confidence interval) 1.49-3.52, *P* < 0.001), dominant (OR = 2.79, 95% CI 1.70-4.59, *P* < 0.001) and heterozygous (OR = 2.88, 95% CI 1.72-4.85, *P* < 0.001) model (Table 2). Additionally, subgroup analysis was conducted by dividing the whole population into three subgroups by atopic types, which include AA, AD, AR groups. No significant association was identified in any genetic models (Table 2).

**Table 2:** Analysis of the effect of TAP1 rs1057141 on the risk of atopic diseases

Genetic model	Population	Studies	Statistics			Heterogeneity			Publication bias	
			OR(95%CI)	Z	*P*	*P*_heterogeneity_	I^2(^%)	Model	*P*_begg_	*P*_egger_
**Allele (G vs. A)**	Overall	10	1.16(0.83–1.63)	0.88	0.378	0.005	62.1	R	0.283	0.160
	AR	4	0.98(0.52–1.86)	0.05	0.963	0.002	79.4	R	1.000	0.699
	AA	2	1.38(0.88–2.16)	1.41	0.158	0.204	38.0	F	1.000	NA
	AD	4	1.26(0.77-2.06)	0.92	0.359	0.198	35.7	F	0.308	0.388
	Asian	7	0.87(0.69-1.08)	1.29	0.196	0.260	22.1	F	0.764	0.591
	African	3	**2.29(1.49-3.52)**	3.79	0.000	0.852	0.0	F	1.000	0.565
**Dominant (GG+AG vs. AA)**	Overall	10	1.22(0.78–1.90)	0.87	0.386	0.000	70.7	R	0.283	0.185
	AR	4	1.01(0.41–2.50)	0.02	0.986	0.000	86.7	R	1.000	0.697
	AA	2	1.60(0.89–2.88)	1.57	0.116	0.273	16.6	F	1.000	NA
	AD	4	1.23(0.76-1.98)	0.83	0.407	0.325	13.5	F	0.308	0.254
	Asian	7	0.86(0.58-1.28)	0.73	0.466	0.051	52.1	R	1.000	0.590
	African	3	**2.79(1.70-4.59)**	4.04	0.000	0.721	0.0	F	1.000	0.937
**Recessive (GG vs. AG+AA)**	Overall	10	1.03(0.57–1.86)	0.09	0.926	0.814	0.0	F	0.466	0.378
	AR	4	0.87(0.34–2.26)	0.28	0.778	0.617	0.0	F	1.000	0.578
	AA	2	1.02(0.31–3.32)	0.03	0.972	0.526	0.0	F	1.000	NA
	AD	4	1.24(0.47–3.28)	0.43	0.667	0.391	0.0	F	1.000	0.815
	Asian	7	0.91(0.46-1.78)	0.29	0.773	0.909	0.0	F	0.260	0.187
	African	3	1.58(0.47-5.31)	0.74	0.457	0.383	0.0	F	1.000	0.339
**Homozygous (GG vs. AA)**	Overall	10	1.08(0.59–1.96)	0.24	0.812	0.834	0.0	F	0.602	0.352
	AR	4	0.88(0.33–2.35)	0.25	0.801	0.809	0.0	F	0.734	0.474
	AA	2	1.17(0.35–3.86)	0.25	0.800	0.483	0.0	F	1.000	NA
	AD	4	1.25(0.47–3.34)	0.45	0.655	0.303	16.4	F	1.000	0.783
	Asian	7	0.89(0.45-1.78)	0.32	0.750	0.970	0.0	F	0.133	0.196
	African	3	2.07(0.60-1.96)	1.16	0.247	0.452	0.0	F	1.000	0.385
**Heterozygous (AG vs. AA)**	Overall	10	0.76(0.76–1.97)	0.85	0.398	0.000	72.2	R	0.283	0.213
	AR	4	1.03(0.38–2.80)	0.06	0.949	0.000	88.4	R	1.000	0.696
	AA	2	1.65(0.94–2.89)	1.75	0.080	0.338	0.0	F	1.000	NA
	AD	4	1.17(0.74–1.85)	0.68	0.500	0.514	0.0	F	0.308	0.148
	Asian	7	0.87(0.56-1.36)	0.60	0.549	0.025	58.4	R	1.000	0.582
	African	3	**2.88(1.72-4.85)**	4.00	0.000	0.565	0.0	F	1.000	0.696

AD, atopic dermatitis; AR, allergic rhinitis; AA, asthma; OR, odds ratio; CI, confidence interval; NA, not applicable; Bold values are statistically significant (*P* < 0.05).

#### TAP1 rs1135216 polymorphism and atopic diseases susceptibility

**Figure 3:**
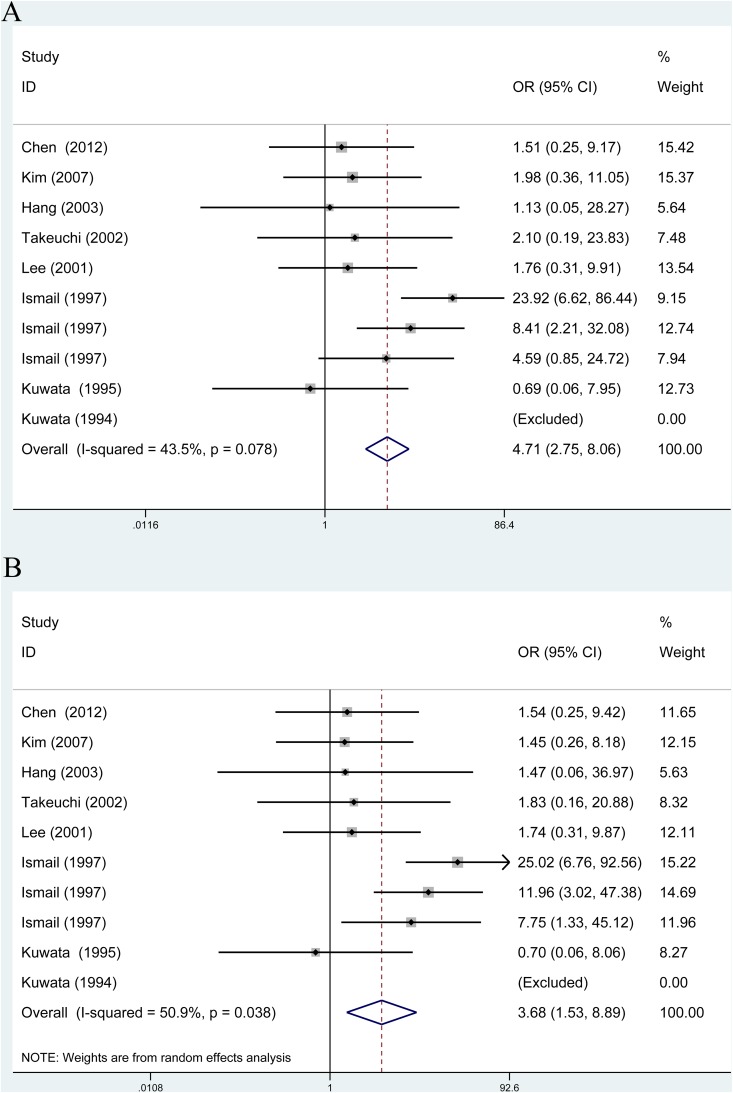
Overall meta-analysis of the relationship between TAP1 rs1135216 polymorphism and atopic diseases risk **A**. Recessive model (GG *vs*. AA+AG); **B**. Homozygote model (GG *vs*. AA).

The results of associations between rs1135216 polymorphism and the risk of atopic diseases were shown in Table 3. A significant increase in the risk of atopic diseases was observed in recessive comparison (OR = 4.71, 95% CI 2.75-8.06, *P* < 0.001) and homozygote comparison (OR = 3.68, 95% CI 1.53-8.89, *P* < 0.001) in overall populations (Figure 3).

**Table 3:** Analysis of the effect of TAP1 rs1135216 on the risk of atopic diseases

Genetic model	Population	Studies	Statistics			Heterogeneity			Publication bias	
			OR(95%CI)	Z	*P*	*P*_heterogeneity_	I^2^(%)	Model	*P*_begg_	*P*_egger_
Allele (G vs. A)	Overall	10	1.52(0.83-2.78)	1.36	0.174	0.000	88.2	R	1.000	0.819
	AR	4	1.22(0.54-2.74)	0.49	0.628	0.000	88.8	R	1.000	0.642
	AA	2	**4.92(1.90-12.69)**	3.29	0.001	0.077	68.1	R	1.000	NA
	AD	4	1.07(0.42-2.70)	0.14	0.885	0.002	79.9	R	0.734	0.604
	Asian	7	0.91(0.62-1.35)	0.46	0.648	0.027	57.8	R	0.764	0.894
	African	3	**5.33(3.67-7.75)**	8.77	0.000	0.318	12.6	F	0.296	0.535
Dominant (GG+AG vs. AA)	Overall	10	1.39(0.76-2.55)	1.07	0.284	0.000	83.8	R	1.000	0.519
	AR	4	1.08(0.45-2.59)	0.18	0.859	0.000	86.6	R	1.000	0.617
	AA	2	**4.10(2.24-7.52)**	4.56	0.000	0.489	0.0	F	1.000	NA
	AD	4	1.04(0.36-3.01)	0.08	0.937	0.003	79.0	R	1.000	0.800
	Asian	7	0.84(0.51-1.39)	0.68	0.500	0.006	67.1	R	0.764	0.881
	African	3	**4.76(2.93-7.73)**	6.29	0.000	0.979	0.0	F	1.000	0.832
Recessive (GG vs. AG+AA)	Overall	10	**4.71(2.75-8.06)**	5.65	0.000	0.078	43.5	F	0.029	0.016
	AR	4	**3.46(1.51-7.93)**	2.94	0.003	0.381	2.3	F	0.734	0.252
	AA	2	7.54(0.41-137.37)	1.36	0.173	0.084	66.5	R	1.000	NA
	AD	4	2.02(0.70-5.81)	1.31	0.191	0.434	0.0	F	0.296	0.372
	Asian	7	1.55(0.67-3.56)	1.03	0.304	0.987	0.0	F	0.133	0.291
	African	3	**12.15(5.54-26.62)**	6.24	0.000	0.267	24.3	F	0.296	0.386
Homozygous (GG vs. AA)	Overall	10	**3.68(1.53-8.89)**	2.90	0.004	0.038	50.9	R	0.251	0.026
	AR	4	**3.52(1.53-8.10)**	2.96	0.003	0.163	41.4	F	1.000	0.269
	AA	2	9.03(0.63-130.17)	1.62	0.106	0.110	60.8	R	1.000	NA
	AD	4	2.28(0.79-6.60)	1.51	0.130	0.240	29.8	F	1.000	0.526
	Asian	7	1.43(0.62-3.32)	0.84	0.400	0.995	0.0	F	0.707	0.534
	African	3	**15.85(6.96-36.08)**	6.58	0.000	0.532	0.0	F	0.296	0.375
Heterozygous (AG vs. AA)	Overall	10	1.11(0.66-1.85)	0.38	0.702	0.000	73.9	R	0.474	0.486
	AR	4	0.92(0.43-1.97)	0.21	0.837	0.002	80.4	R	0.734	0.670
	AA	2	**2.18(1.11-4.31)**	2.25	0.024	0.190	41.8	F	1.000	NA
	AD	4	1.00(0.36-2.80)	0.01	0.996	0.006	75.7	R	1.000	0.845
	Asian	7	0.81(0.48-1.36)	0.80	0.425	0.004	68.3	R	0.764	0.899
	African	3	**2.54(1.44-4.46)**	3.24	0.001	0.235	30.9	F	1.000	0.876

**Figure 4:**
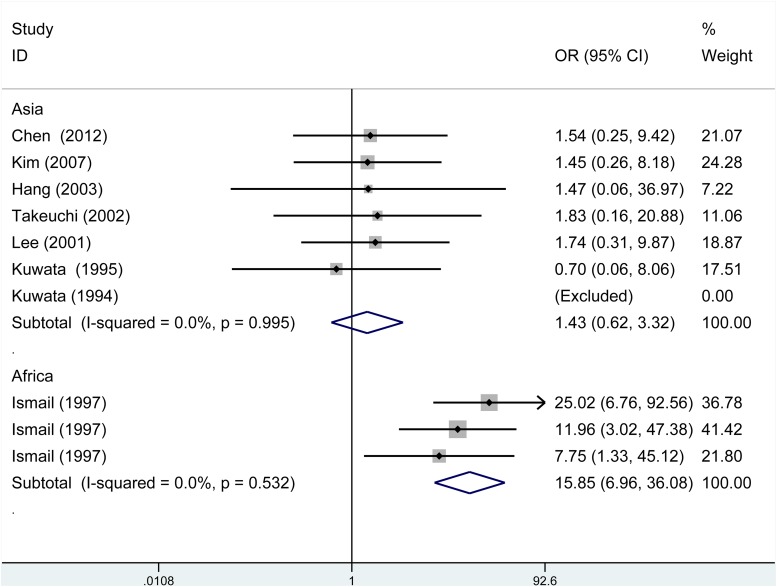
Subgroup analysis of the relationship between TAP1 rs1135216 polymorphism and atopic diseases stratified by the ethnicity of study population I^2^ and Pheterogeneity for the outcomes of the homozygous comparison in Asians (I^2^ = 0%, *P*_h_=0.995) and Africans (I^2^=0%, *P*_h_=0.532).

From subgroup analysis by ethnicity, it demonstrated a significant association of TAP1 rs1135216 polymorphism and atopic diseases in Africans in all five genetic models (G versus A: OR = 5.33, 95% CI 3.67-7.75, *P* < 0.001; AG/GG versus AA: OR = 4.76, 95% CI 2.93-7.73, *P* < 0.001; GG versus AG/AA: OR = 12.15, 95% CI 5.54-26.62, *P* < 0.001; GG versus AA: OR = 15.85, 95% CI 6.96-36.08, *P* < 0.001; AG versus AA: OR = 2.54, 95% CI 1.44-4.46, *P* = 0.001) but not in Asians (Table 3). Interestingly, when stratified by ethnicity, heterogeneity disappeared in Asians (I^2^ = 0%, P_h_ = 0.995) and Africans (I^2^ = 0%, P_h_ = 0.532) in homozygous comparison (Figure 4). Subgroup analysis by atopic types indicated a significant association in AA in the allele (OR = 4.92, 95% CI 1.90-12.69, *P* = 0.001), dominant (OR = 4.10, 95% CI 2.24-7.52, *P* < 0.001) and heterozygous (OR = 2.18, 95% CI 1.11-4.31, *P* = 0.024) models and AR in the recessive (OR = 3.46, 95% CI 1.51-7.93, *P* = 0.003) and homozygous (OR = 3.52, 95% CI 1.53-8.10, *P* = 0.003) models (Table 3).

#### Publication bias and sensitivity analysis

**Figure 5:**
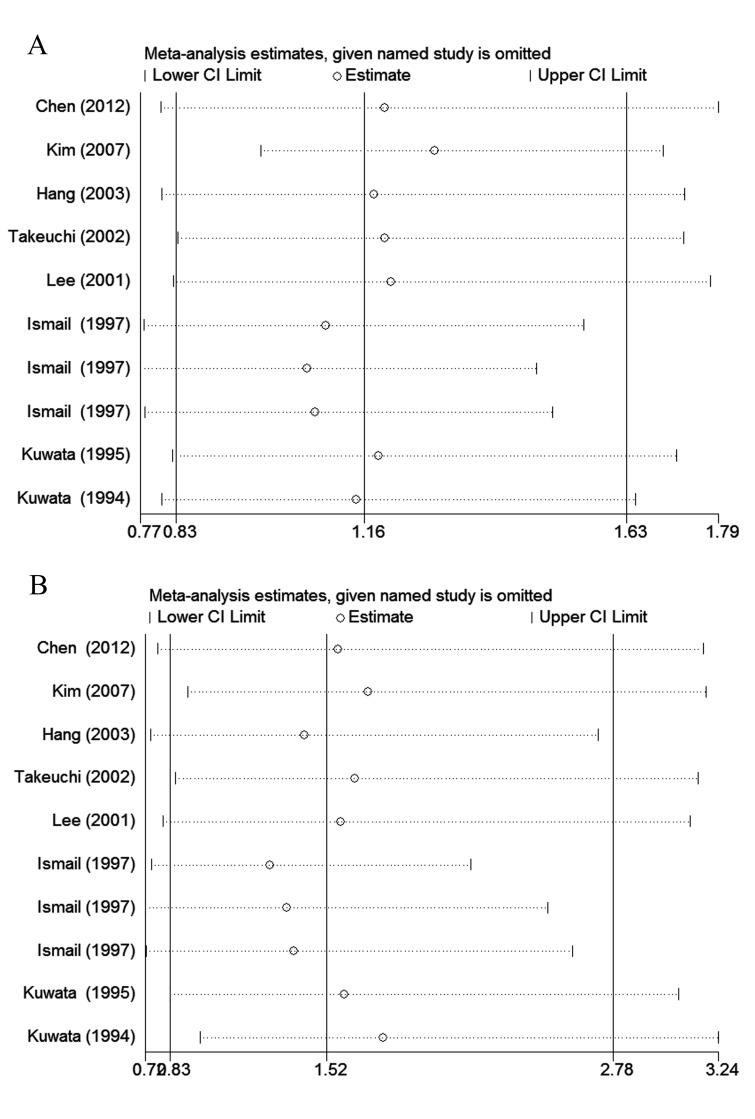
The impact of individual studies on the overall OR in allele model (G *vs.* A) **A.** rs1057141; **B.** rs1135216.

AD, atopic dermatitis; AR, allergic rhinitis; AA, asthma; OR, odds ratio; CI, confidence interval; NA, not applicable; Bold values are statistically significant (*P* < 0.05).

With the use of Begg's funnel plot and quantitative Egger's test, publication bias was assessed and no obvious publication bias was found for the association between rs1057141 and atopic diseases risk (Table 2). Symmetrical funnel plots were obtained in all the genetic models. As for rs1135216, quantitative Egger's test of the included studies showed potential publication bias in recessive comparison (*P*_egger_ = 0.016) and homozygote comparison (*P*_egger_ = 0.026) (Table 3). However, when stratified by ethnicity, such publication bias disappeared in both Asian (recessive: *P*_egger_ = 0.291; homozygous: *P*_egger_ = 0.534) and African (recessive: *P*_egger_ = 0.386; homozygous: *P*_egger_ = 0.375) groups (Table 3). Result from trim-and-fill analysis, which was to investigate the effect of missing trials, showed “no trimming performed; data unchanged”. In addition, sensitivity analysis which evaluated the impact of individual study on overall ORs showed that exclusion of any study made no significant difference (Figure 5).

## DISCUSSION

Our study for the first time assessed the relationships between TAP1 rs1057141 and rs1135216 polymorphism and atopic diseases risk through quantitative meta-analysis of the published case-control studies. The results showed that rs1135216 polymorphism might have a significant association with atopic diseases risk.

Allergic diseases are frequent chronic diseases and recognized as a group of immune-mediated disorders [17]. Progress of allergic symptoms could start with atopic dermatitis that could be accompanied with sensitization to aeroallergen during early childhood, followed by asthma and allergic rhinitis during later childhood or adult life [2, 18]. Epidemiological studies have demonstrated the atopic diseases have developed as an epidemic disease through the last decades. However, pathogenesis, natural course and the underlying mechanisms of atopic diseases is still unclear. [19]. Etiology of AD includes alterations in skin barrier function, immune dysregulation and exposure to environmental, which could ultimately lead to the classic skin manifestation of AD [20]. Environmental allergens could cross a dysfunctional skin barrier, which will activate immune system and elicit AD symptoms. Antigen presentation, which is required for priming the immunologic response to external antigen is regulated by HLA molecules. HLA are encoded within MHC located on chromosome 6 [21] which hosts hundreds of genes that code for cell-surface antigens and therefore affect immune system function [21].

TAP gene is located within the MHC class II region of chromosome 6 between the HLA-DP and HLA-DQ loci. The TAP protein is critical for the processing and presentation of intracellular antigens [22]. Though most studies on the influences of TAP1 on atopic diseases susceptibility reported consistent findings, contradictory results have been observed from replicating studies of different ethnic populations. TAP1 rs1135216, as a common TAP1 gene polymorphism, is associated with atopic diseases [5–7, 9, 10]. Substitution of the 637^th^ amino acid aspartate by glycine in TAP1 protein resulted from the replacement of the 2120^th^ base adenine by guanine in TAP1 rs1135215 compared with that of TAP1 gene seems responsible for the increased atopic diseases risk. From the 10 studies analyzing rs1135216 in exon 10, 4 studies found significant association between rs1135216 and atopic diseases, which was absent from the other 6 studies.

The present meta-analysis systematically evaluated the association between rs1057141 and rs1135216 polymorphisms and atopic diseases susceptibility. Positive association between TAP1 rs1135216 polymorphism and increased atopic diseases risk was observed and patients with the GG genotype may be susceptible to atopic diseases. High heterogeneities were observed in the allele, dominant and heterozygous models. However, when stratified by ethnicity, heterogeneity disappeared in Asians and Africans. Difference between Asians and Africans indicated that rs1135216 polymorphism might be ethnicity-specific. Sub-population analysis revealed association between different genotypes and Africa population. The atopic diseases risk increased significantly in Africa populations using allelic, dominant, recessive, homozygous and heterozygous genetic models. As to rs1057141, increased risk of atopic disease in the allelic, dominant and heterozygous model was found in African population. Additionally, with sensitivity analysis indicating our data stable and robust, our results may assist the establishment of personal therapy in atopic diseases.

A few limitations presented in this meta-analysis should be considered if the data are to be used further. First, different research methods may increase the heterogeneity of these studies. Second, only Asian and African populations are included in the current analysis, and therefore studies on ethnicities such as Caucasians are needed for further investigation. Additionally, the heterogeneity present in some genetic models also posed as limitation of this study. To overcome the limitation present in this study, large-scale studies are required to validate the significance of our conclusions.

In summary, this meta-analysis supported evidence that rs1135216 polymorphism may confer susceptibility to atopic diseases in the overall population and especially among Africans.

## MATERIALS AND METHODS

Conduction of meta-analysis was based on the PRISMA guidelines [23].

### Search strategy and selection criteria

Databases including PubMed, EMBASE and Web of Science (last updated on July, 2017) were used as the source for identifying published investigations on the relationship between TAP1 polymorphism and atopic risk. Time was not limited for the search of the eligible literature. Keywords used for searching the literature include: ‘transporter associated with antigen processing 1’,‘TAP1’, ‘polymorphism’, ‘variant’, ‘genotype’, ‘allele’, ‘dermatitis’, ‘rhinitis’, and ‘asthma’. Besides, to identify further eligible articles, reference lists of all relevant articles were reviewed manually.

Criteria used for selection of eligible studies include: (1) being a unrelated case-control design study; (2) include investigation of the relationship between TAP1 polymorphisms and atopic dermatitis, allergic rhinitis, and/or asthma; (3) including sufficient data to calculate the pooled ORs with 95% CI [24].

### Data extraction and quality assessment

The types of data extracted from the included studies are: first author, publication year, country, ethnicity, genotype methods, the number of cases and controls, distributions of genotypes and alleles in atopic patients and control subjects, and the probability value of HWE in control group. Data extraction was carried out independently by Liu and Chen who also performed study quality assessment. Disagreements were solved by discussion.

Scoring system was established by incorporating traditional epidemiological considerations together with atopic genetic issues recommended by Thakkinstian to assess study quality [25]. Six perspectives were evaluated to assess the study quality: representativeness of samples, ascertainment of samples, genotyping methods, association assessment, response rate and HWE of the control groups (Supplementary Table 1) [26]. The scores were set ranging from 0 (worst) to 15 (best). Any disagreement aroused between the previous mentioned two researchers was adjudicated by a third researcher (Qi). Any study with a quality score of ≤ 4 was considered to be low quality and excluded for further analysis [27].

### Statistical analysis

Statistical analysis was performed with StataMP 14 (StataCorp, College Station, TX). Five genetic models including the allelic (G versus A), dominant (AG+GG versus AA), recessive (GG versus AG+AA), homozygous (GG versus AA) and heterozygous (AG versus AA) models were adopted [28]. The relationships between TAP1 gene polymorphism and atopic diseases amongst different models were compared using OR and its corresponding 95% CI. Chi-squared-based Q-test was employed to evaluate the heterogeneity between the individual studies with *P* < 0.1 considered as significant. In the presence of heterogeneity among individual studies, estimation of pooled OR was conducted utilizing a random-effect model. Otherwise, a fixed-effect model was applied. The pooled OR was determined by Z-test, and OR with *P* < 0.05 was considered as significant [29]. To confirm the validity of HWE, Genotype distributions in the control subjects were tested with χ^2^ test, and significance was set at *P* < 0.05. Subgroup analysis by ethnicity and atopic types was performed to investigate possible causes of heterogeneity. Influence of individual study on the overall analysis outcome was assessed by sensitivity analysis, in which the results by stability was tested by excluding each study in sequence to examine their impact on the test of heterogeneity. To determine whether there existed publication bias, Funnel plots, Begg's test and Egger's test were applied. In the case of presence of publication bias, the trim and fill approach was applied to adjust the meta-analysis results by imputing data from presumed missing studies [30].

## SUPPLEMENTARY MATERIALS TABLE


