# Supplementary material for: Associations of TAP1 genetic polymorphisms with atopic diseases: asthma, rhinitis and dermatitis
Source: Oncotarget. 2017 Dec 20;9(2):1553–62. doi: 10.18632/oncotarget.23458 (PMC5788581; doi:10.18632/oncotarget.23458)
Supplement: Supplementary file 1 [file oncotarget-09-1553-s001.pdf]

Associations of TAP1 genetic polymorphisms with atopic diseases: asthma, rhinitis and dermatitis

SUPPLEMENTARY MATERIALS

Supplementary Table 1: Scale for quality assessment of molecular association studies of atopic diseases Total scores were set ranging from 0 (worst) to 15 (best). Any study with a quality score of ≤ 4 was considered to be low quality and excluded for further analysis

Criteria	Score
**Representativeness of cases**	
Consecutive/randomly selected from case population with clearly defined sampling frame	2
Consecutive/randomly selected from case population without clearly defined sampling frame or with extensive inclusion/exclusion criteria	1
No method of selection described	0
**Representativeness of controls**	
Controls were consecutive/randomly drawn from the same sampling frame (ward/community) as cases	2
Controls were consecutive/randomly drawn from a different sampling frame as cases	1
Not described	0
**Ascertainment of cases**	
Clearly described objective criteria for diagnosis of asthma, rhinitis and dermatitis	2
Diagnosis of asthma, rhinitis and dermatitis by patient self-report or by patient history	1
Not described	0
**Ascertainment of controls**	
Controls were tested to screen out asthma, rhinitis and dermatitis	2
Controls were subjects who did not report asthma, rhinitis and dermatitis; no objective testing	1
Not described	0
**Genotyping examination**	
Genotyping done under "blinded" condition	1
Unblinded or not mentioned	0
**Hardy-Weinberg equilibrium**	
Hardy-Weinberg equilibrium in control group	2
Hardy-Weinberg disequilibrium in control group	1
No checking for Hardy-Weinberg equilibrium	0
**Association assessment**	
Assess association between genotypes and asthma, rhinitis and dermatitis with appropriate statistics and adjustment for confounders	2
Assess association between genotypes and asthma, rhinitis and dermatitis with appropriate statistics without adjustment for confounders	1
Inappropriate statistics used	0
**Response rate**	
Response rates for both groups are the same,i.e., to within 5%	2
Response rates are different, between 5% and 10%	1
Response rates are more than 10% different, or no mention of response rates	0
